# A comprehensive review and bibliometric analysis on collaborative robotics for industry: safety emerging as a core focus

**DOI:** 10.3389/frobt.2025.1605682

**Published:** 2025-09-12

**Authors:** Aida Haghighi, Morteza Cheraghi, Jérôme Pocachard, Valérie Botta-Genoulaz, Sabrina Jocelyn, Hamidreza Pourzarei

**Affiliations:** 1 School of Occupational and Public Health, Faculty of Community Services, Toronto Metropolitan University, Toronto, ON, Canada; 2 Department of Mechanical, Industrial and Mechatronics Engineering, Toronto Metropolitan University, Toronto, ON, Canada; 3 National Education, Engineering Sciences, Lycée Henri Loritz, Nancy, France; 4 INSA Lyon, Université Lumière Lyon 2, Universite Claude Bernard Lyon 1, Université Jean Monnet Saint-Etienne, DISP UR4570, Villeurbanne, France; 5 Institut de Recherche Robert-Sauvé en Santé et en Sécurité du Travail (IRSST), Montréal, QC, Canada; 6 Systems Engineering Department, École de technologie supérieure – ETS, Montreal, QC, Canada

**Keywords:** collaborative robotics, cobot, scoping review, bibliometric analysis, cobotics research trends, safety

## Abstract

Research organizations and academics often seek to map the development of scientific fields, identify research gaps, and guide the direction of future research. In cobot-related research, the scientific literature consulted does not propose any comprehensive research agenda. Moreover, cobots, industrial robots inherently designed to collaborate with humans, bring with them emerging issues. To solve them, interdisciplinary research is often essential (e.g., combination of engineering, ergonomics and biomechanics expertise to handle safety challenges). This paper proposes an exhaustive study that employs a scoping review and bibliometric analysis to provide a structured macro perspective on the developments, key topics, and trends in cobot research for industry. A total of 2,195 scientific publications were gained from the Web of Science database, and a thorough selection process narrowed them down to 532 papers for comprehensive analysis. Descriptive statistics were employed to analyze bibliometric measures, highlighting publication trends, leading journals, the most productive institutions, engaged countries, influential authors, and prominent research topics. Co-authorship and bibliographic couplings were also examined. Through a co-occurrence analysis of terms, the content and research objectives of the papers were systematically reviewed and lead to a univocal categorization framework. That categorization can support organizations or researchers in different cobotics (collaborative robotics) fields by understanding research developments and trends, identifying collaboration opportunities, selecting suitable publication venues, advancing the theoretical and experimental understanding of automatic collaborative systems, and identifying research directions and predicting the evolution of publication quantity in cobotics.

## 1 Introduction

Human-Robot collaboration is a current industrial trend following the development of new enabling technologies in the scope of Industry 4.0 (Bortolini et al., 2017; Rosin et al., 2020; Vysocky and Novak, 2016). Humans and robots can work jointly if sufficient safety is guaranteed, leading to the concept of cobots first introduced in (Colgate et al., 1996). Initially, that word “cobot” referred to intrinsically passive manipulators (Colgate et al., 1996). Nowadays, the term “cobot” refers to active manipulators that are industrial robots in the sense of ISO 10218-1:2025 (ISO, 2025a), inherently designed to physically interact with humans or share a same workspace, thanks to one or more of the three following methods: 1) hand-guided control (HGC), 2) speed and separation monitoring (SSM), or 3) power and force limiting (PFL) as described in ISO 10218-2:2025 (ISO, 2025b). That interaction or that space sharing is possible if the risks assessed, associated with the collaborative application, are deemed acceptable for the humans in the vicinity of the cobot. When that collaboration is possible, one can benefit from the advantages of both, thereby transcending the conventional division of labor that often mandates robots to be confined in safety cages, away from human workers. In a collaborative system, humans contribute flexibility, intelligence, cognitive skills, and the capacity to tackle unforeseen challenges, whereas cobots excel in executing repetitive and monotonous tasks with accuracy, agility, and strength (Paliga, 2022). This new technology, cobot, presents an opportunity to reinvent manufacturing systems, leading to better efficiency while improving working conditions (Schmidtler et al., 2015), and possibly even offering benefits for environmental considerations. For example, Alvarez-de-los-Mozos et al. (2020) utilized a cobot for recycling electrical and electronic waste.

As shown in the two subsequent paragraphs below, various papers have been dedicated to reviewing and discussing the existing literature on human-robot interaction in general or on cobots specifically. The aim of these papers is to provide valuable insights into specific topics such as safety in workspaces where humans and cobots interact with each other (Bi et al., 2022; Bi et al., 2021; Bogue, 2017; Broum and Simon, 2020; Chemweno et al., 2020; Kumar et al., 2020; Li et al., 2023; Valori et al., 2021), terminology in the safety of cobotics (Vicentini, 2020), human awareness during collaboration (Grushko et al., 2021), physical and cognitive ergonomics in cobotic workstations (Cardoso et al., 2021), collaborative robotics applications (Montini et al., 2024; Liu et al., 2024a), developments in gripper technologies for cobots (Bogue, 2016a), task planning and programming (El Zaatari et al., 2019; Tsarouchi et al., 2016), designing workplaces where humans and cobots interact with each other (Simoes et al., 2022), and learning methodologies for human-robot collaboration (Mukherjee et al., 2022).

For example, Gualtieri et al. (2021a) conducted a systematic literature review on both safety and ergonomics in cobotics for industry. Similarly, Patil et al. (2023) systematically reviewed safety and ergonomics in cobotics and classified the literature into four sub-categories: contact avoidance and contact detection and mitigation for safety category and physical ergonomics and cognitive ergonomics for ergonomics category. Storm et al. (2022) reviewed safety along with the mental health and wellbeing of workers near cobots. Lu L. et al. (2022) utilized a systematic literature review methodology to identify robot-related factors affecting cobot-related workers’ mental stress or safety awareness. They discussed methods to measure mental stress and safety awareness during human-robot collaboration. Berx et al. (2022a) reviewed literature on cobotics to identify and classify risk factors in human-robot collaboration. Vicentini (2021) provided an extensive review on cobotics, focusing on safety and task planning. Also, valuable information on sensors and actuators for cobots can be found in Bogue et al. (2015), Ogenyi et al. (2021). Villani et al. (2018) carried out a review on cobots, focusing on issues related to physical and cognitive interaction. The issues related to safety, robot programming, sensing technologies for human-robot interaction, and industrial applications of cobotics are explicitly discussed in that paper. While those studies focused on specific topics or challenges in cobotics, the current paper covers scientific articles dealing with all kinds of topics and challenges related to cobotics.

Hentout et al. (2019) studied literature on human-robot interaction in cobotics for industry from 2008 to 2017. They attempted to classify the content of the published works, resulting in a stimulating classification with seven categories, each broken down into several subcategories. Similarly, Matheson et al. (2019) did a practical study on human-robot collaboration in manufacturing from 2009 until 2018, including a remarkable cobot market analysis. They classified the publications into three topics categories (i.e., productivity, safety, human-robot interaction), pointed out some limitations of their equivocal categories. Proia et al. (2022) carried out a systematic review of the control techniques used in cobotics. They classified research works into three main categories: safety, ergonomics, and efficiency. They further discussed and categorized them into sub-categories within each main category to highlight the types of control systems. In addition, Borregan-Alvarado et al. (2024) proposed a model to identify and predict of research topics in collaborative robotics and human-robot interaction technologies. That model is based on scientific articles on human-robot interaction for the 2020–2021 period. The current paper not only covers a broader and more recent time frame (1996–2022), but also provides a clear and consistent univocal categorization framework based on key topics identified through an in-depth bibliometric analysis. Also, given the need to support cobotics researchers in identifying the most active research areas, and relevant journals for publication, as well as opportunities for collaboration to tackle the emerging challenges associated with cobotics, this paper performs a bibliometric analysis on two levels: (1) all cobot-related publications, and (2) specific research fields within cobotics.

As discussed above, the growing attention towards cobots has motivated researchers to generate various review papers in cobotics. Although the previous studies have offered valuable insights, there is a lack of a comprehensive quantitative and qualitative analysis of the current state-of-the-art in cobotics. To address this gap, the present study aims to carry out a thorough review and bibliometric analysis of studies dedicated to cobotics for industry. In summary, this study offers the following specific novel contributions:

(i) Covering an extensive timeframe, it reviews over 25 years of scientific literature since the term “cobot” was first coined in 1996, capturing the evolution and maturation of the field.
(ii) It includes scientific literature dealing with all kinds of topics and challenges related to cobotics.
(iii) It conducts an in-depth bibliometric analysis of cobotics-related literature for industry on two levels: (1) all cobot-related publications, and (2) specific research fields within cobotics. This analysis examines publication trends, leading journals, productive institutions, engaged countries, influential authors, and emerging topics, serving as a critical reference for academics and industry professionals (e.g., engineers) navigating the rapidly evolving field of cobotics.
(iv) It identifies key research directions and predicts the trajectory of publication growth in this domain.
(v) It introduces a clear and consistent univocal categorization framework encompassing six main research topics and 25 subcategories, providing a valuable roadmap for researchers exploring diverse aspects related to cobotics, including safety in collaborative applications, cobot deployment in industrial settings, task optimization between humans and robots, communication between humans and cobots, and cobot actuating systems.

By providing both a structured understanding of existing research and insights into future trends, this work equips scholars and practitioners with the necessary guidance to drive advancements in cobotics.

Besides this introductory section, this paper is organized as follows: the methodology is presented in Section 2, while the results and discussions of quantitative and qualitative analysis of cobot-related research for industrial settings derived from the literature are presented in Sections 3, 4. The final section presents the conclusions and agenda for future research.

## 2 Methodology

The Preferred Reporting Items for Systematic Reviews and Meta-Analyses extension for Scoping Reviews (PRISMA-ScR) (Tricco et al., 2018) guided this scoping review in the following steps.

### 2.1 Research questions

This paper aims to address the following main research questions: (i) What are the predominant research interests from the first cobot, i.e., 1996, to the end of 2022, and how can these be systematically classified?; (ii) which research topics in cobotics have garnered the most attention, and which studies and journals are considered most influential in this field?; (iii) who are the leading authors and which countries have made the most significant contributions to cobotics research?; (iv) how has the publication quantity in cobotics evolved over the years, and what trends can be predicted for future research output in this field?; and (v) what are the existing research gaps in cobotics, and what future research directions can be identified through a comprehensive scoping review and bibliometric analysis?

### 2.2 Identifying relevant studies

**FIGURE 1:**
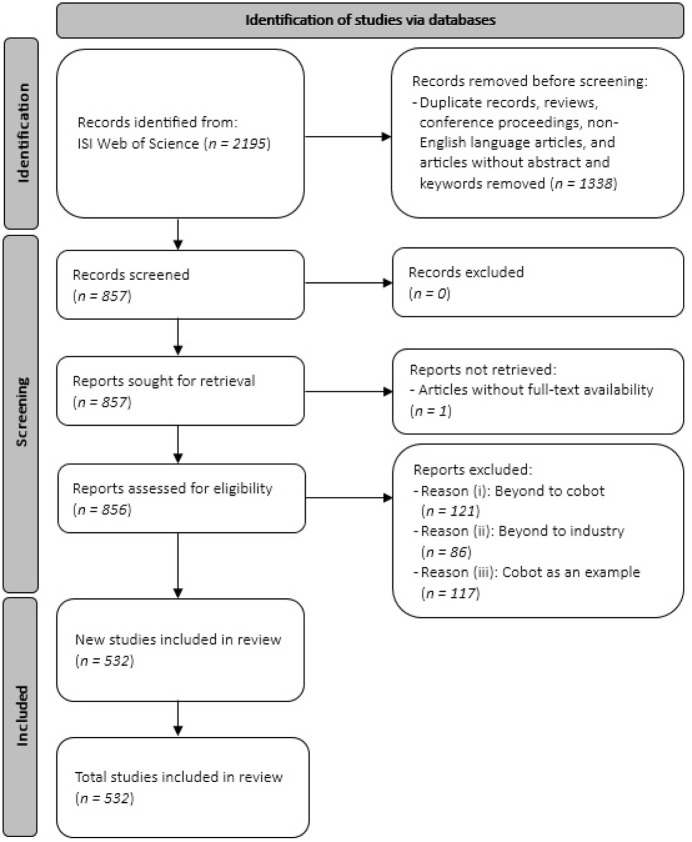
PRISMA-ScR process for selecting the literature.

First, a comprehensive search strategy was formulated to access a diverse array of articles for consideration. This search was carried out in the ISI Web of Science (WoS), recognized as one of the premier repositories in research, covering the earliest date available in the database up to, and including, December 2022. The search string used was “collaborative robot*” OR cobot * in either the title, abstract, or author keywords fields. Due to the extensive nature of the research conducted, our objective was to identify as many relevant papers (including journal articles, reviews, and conference proceedings) as possible, focusing solely on cobots, totaling 2,195 records. That two-word search string was chosen after trying broader strategies leading to more records. Those broader strategies, including for example, the additional terms OR “human robot interaction” OR HRI multiplied the number of records by at least 40. However, a significant portion of those records was not related to cobots, as per its meaning presented in Section 1. Indeed, research on human-robot interaction or collaboration is also dealt with in the literature for industrial robots that are not inherently designed to interact with humans or for service robots for example. Adding other keywords made the amount of articles to screen impossible to assess humanly in a timely manner by the research assistants, due to limited staff, as well as time and budget constraints. Because of that, and to minimize the number of papers not related to cobots in an industrial context, the paper focuses only on the two-keyword strategy. Analyzing the papers started in 2023 and finished in 2024. The writing of the paper followed in 2025. Figure 1 illustrates the process of the PRISMA-ScR guideline for selecting the relevant literature and the number of papers handled at each part for the chosen strategy.

### 2.3 Selecting studies

As can be seen in Figure 1, for conducting a scoping review, first, the focus was narrowed only to journal articles with abstracts, keywords, and DOIs, resulting in 857 documents. One record out of these 857 documents was excluded due to the reason of without full-text availability. Then, a screening process was used in order to ensure the exclusion of irrelevant articles. To minimize threats to validity, one team member conducted the screening process while another independently validated their work. Any disagreements during the screening were resolved through discussion between the two team members to reach a consensus and make a final decision. This screening process resulted in the exclusion of 323 papers, based on three specific exclusion criteria (see Figure 1):

(i) Beyond to cobot: 121 papers matched the search keywords but had a different definition from that provided in the introduction. Interestingly, among these articles, 20 focused on various types of robots (often aerial and/or terrestrial robots, and swarms of robots) working collaboratively, but without human.
(ii) Beyond to industry: 86 articles addressed cobots but in fields outside of industry. For example, among these articles, 54 were related to the medical field.
(iii) Cobot as an example: 117 articles occasionally mentioned cobots alongside other Industry 4.0 technologies or used cobots as a testing device for other equipment.

After this screening process, 532 articles were validated for final analysis in this research.

### 2.4 Charting the data

In this paper, MS Excel and VOSviewer were used for the bibliometric analysis of the 532 retrieved papers. VOSviewer employs the VOS (Visualization of Similarities) mapping method to create networks where the distances between items indicate their degree of similarity. The VOS clustering technique categorizes topics into distinct clusters, each marked with a different color (van Eck and Waltman, 2010; van Eck and Waltman, 2007). These techniques allow for the analysis of papers in terms of co-authorship, co-occurrence of terms in the titles, abstracts, and keywords, as well as the bibliographic couplings of research institutions, countries, or publication sources. Thanks to these features, VOSviewer has been widely applied in the bibliometric analysis in various robotics fields (Wu et al., 2024; Wang J. et al., 2023; Mudhivarthi and Thakur, 2022; Long et al., 2024; Liu and Son, 2024; Li W-S. et al., 2021; Chu et al., 2021). Based on the key topics identified through the bibliometric analysis, a univocal categorization framework was proposed. Two team members reviewed the papers, classifying each to a category based on its main research objective. Any discrepancies were resolved through discussion.

### 2.5 Synthesizing and reporting the results

The research topic categories were formed and organized by reviewing the papers and their research topics. Furthermore, MS Excel and VOSviewer were used again for bibliometric analysis on each category to gain more specific results for each research topics.

## 3 Bibliometric analysis–Results and discussions

In this section, we present and discuss the results from the bibliometric analysis of the 532 retrieved papers. In general, the visualizations are interpreted as follows: the size of the spheres and the font of the labels represent the number of occurrences, the colors represent clusters (i.e., a set of closely related items), and the distance between two spheres indicate their relatedness and similarity.

### 3.1 Publication trends

**FIGURE 2:**
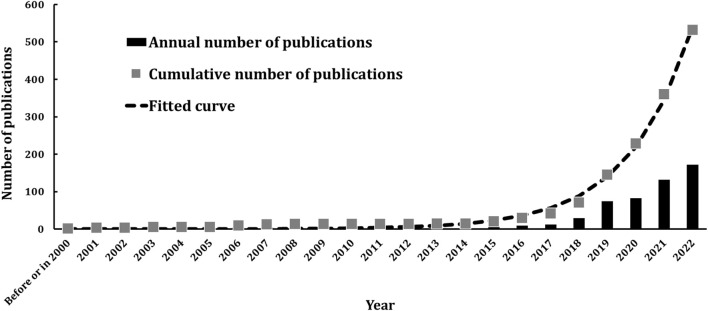
Annual and cumulative number of publications.

The number of publications is an important indicator for measuring the development trends within a research domain. By analyzing the number of publications over time, one can easily infer research activity levels and trends, which in turn allows for the estimation of future activity levels (Yang et al., 2019). Figure 2 presents the annual and cumulative number of publications focused on the topic of cobotics for industry. It shows that there were only 14 publications before or in 2015. This period can be considered the initial stage, where little research explicitly focused on cobot in industrial settings. Since 2016, the number of publications in this field has increased significantly. Furthermore, the cumulative number of publications approximately follows an exponential growth pattern, as calculated by Equation 1:

$$Cumulativenumberofpapers\left(n\right)={a_{1}\times e}^{\left(a_{2}\left(n-2000\right)\right)}$$ (1)

where n is the year for which we want to predict the cumulative number of papers, and $a_{1}$ and $a_{2}$ are coefficients estimated to be 0.02632 and 0.4514, respectively. Therefore, the cumulative number of papers in this field in a specific year can be calculated using Equation 2:

$$Cumulativenumberofpapers\left(n\right)={0.02632\times e}^{\left(0.4514\left(n-2000\right)\right)}$$ (2)

The Coefficient of Determination ( $R^{2}$ ) is used to measure the fitting degree and can be calculated using Equation 3:

$$R^{2}=1-\frac{\sum_{n=1}^{N}{\left(\begin{array}{c} Actualcumulativenumberofpapers\left(n\right)\\ -Predictedcumulativenumberofpapers\left(n\right) \end{array}\right)}^{2}}{\sum_{n=1}^{N}{\left(\begin{array}{c} Actualcumulativenumberofpapers\left(n\right)\\ -Meanofactualcumulativenumberofpapers\left(n\right) \end{array}\right)}^{2}}$$ (3)

where N is the number of observations. An $R^{2}$ value closer to 1 indicates a better fit of the regression model, while a value closer to 0 indicates a poorer fit. Also, to measure the prediction error, the Root Mean Squared Error ( RMSE ) is used, as shown in Equation 4:

$$RMSE=\sqrt{\frac{1}{n}\sum_{n=1}^{N}{\left(\begin{array}{c} Actualcumulativenumberofpapers\left(n\right)\\ -Predictedcumulativenumberofpapers\left(n\right) \end{array}\right)}^{2}}$$ (4)

The model demonstrates a high goodness-of-fit with an $R^{2}$ value of 0.995, indicating that the cumulative number of published papers in this field is growing exponentially, and that continued development in cobotics research is anticipated. The RMSE is 9.15, suggesting that the average prediction error is around 10 papers. The predicted cumulative number of publications is listed in Table 1. The number of publications is expected to reach approximately 2000 by 2025, and about 20000 by 2030.

**TABLE 1:** Predicted cumulative number of publications.

Year	Cumulative number of articles	Predicted cumulative number of articles	Year	Cumulative number of articles	Predicted cumulative number of articles
2000	1	0.03	2016	29	36.05
2001	3	0.04	2017	41	56.62
2002	3	0.06	2018	70	88.92
2003	5	0.10	2019	145	139.65
2004	5	0.16	2020	228	219.33
2005	5	0.25	2021	360	344.46
2006	9	0.39	2022	532	540.98
2007	12	0.62	2023	-	849.61
2008	13	0.97	2024	-	1,334.32
2009	13	1.53	2025	-	2095.56
2010	13	2.40	2026	-	3,291.09
2011	13	3.77	2027	-	5,168.69
2012	13	5.93	2028	-	8,117.47
2013	14	9.31	2029	-	12748.56
2014	14	14.62	2030	-	20021.74
2015	20	22.96	2031	-	31444.33

### 3.2 Geographical and institutional distribution and cooperation

The geographical span of a research field indicates its versatility and global popularity. The more institutions/countries involved, the more significant the research field. This aspect is explored in the following subsections.

#### 3.2.1 Contributing countries

**FIGURE 3:**
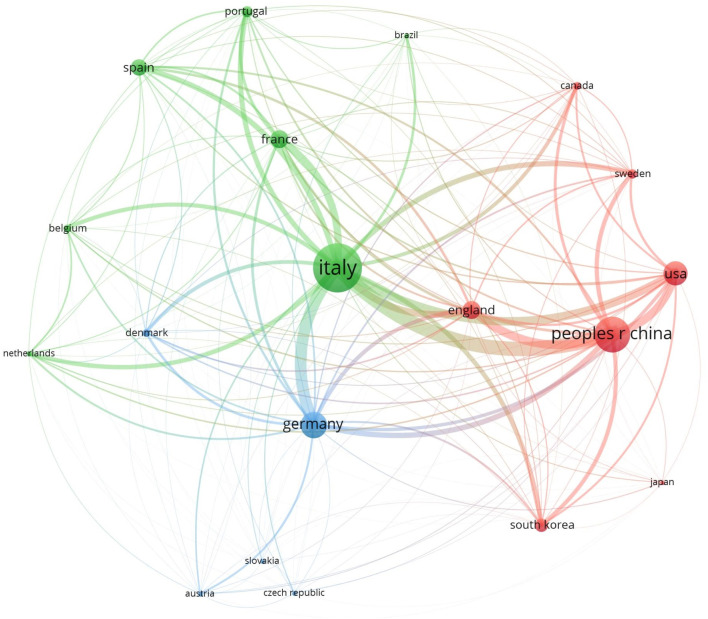
Bibliographic coupling among countries with at least 10 published papers.

The 532 retrieved papers come from 59 distinct countries (see Table 2; Figure 3). Figure 3 illustrates the bibliographic coupling among these countries. Bibliographic coupling occurs when publications from two countries reference works from a third country. Each country is represented by a sphere, with the size of the sphere and its label indicating the magnitude of the contribution; larger spheres and labels denote greater contributions. The thickness of the arcs connecting spheres represents bibliographic connections while the colors indicate collaboration clusters of countries.

**TABLE 2:** Top contributing countries to the field of cobotics for industry.

Rank	Country	Number of articles	% of articles	Number of citations	Average number of citations	Population, (millions)	Gross domestic product, GDP, (millions USD)	GDP *per capita*, (thousand USD)	Number of articles *per capita*, NAC, (per million)	Number of articles *per capita* GDP, NAG, (per thousand USD)
1	Italy	105	19.74	2,970	28.29	58.76	2254851	38.373	1.79	2.74
2	PRC	77	14.47	1,417	18.40	1,410.71	17794781	12.614	0.05	6.10
3	Germany	57	10.71	1,169	20.51	84.48	4456081	52.745	0.67	1.08
4	USA	52	9.77	1,692	32.54	334.91	27360935	81.695	0.16	0.64
5	France	39	7.33	1,197	30.69	68.17	3030904	44.460	0.57	0.88
6	England	38	7.14	969	25.50	68.35	3340032	48.866	0.56	0.78
7	Spain	35	6.58	804	22.97	48.37	1580694	32.677	0.72	1.07
8	South Korea	29	5.45	354	12.21	51.71	1712792	33.121	0.56	0.88
9	Portugal	24	4.51	536	22.33	10.52	287080	27.275	2.28	0.88
10	Sweden	21	3.95	530	25.24	10.53	593267	56.305	1.99	0.37
11	Belgium	18	3.38	328	18.22	11.82	632216	53.475	1.52	0.34
12	Canada	18	3.38	395	21.94	40.09	2140085	53.371	0.45	0.34
13	Denmark	18	3.38	633	35.17	5.94	404198	67.967	3.03	0.26
14	Slovakia	11	2.07	103	9.36	5.42	132793	24.470	2.03	0.45
15	Austria	11	2.07	114	10.36	9.13	516034	56.506	1.20	0.19
16	Brazil	11	2.07	133	12.09	216.42	2173665	10.043	0.05	1.10
17	Japan	11	2.07	230	20.91	124.51	4212945	33.834	0.09	0.33
18	Netherlands	11	2.07	254	23.09	17.87	1118124	62.536	0.62	0.18
19	Czech republic	10	1.88	66	6.60	10.87	330858	30.427	0.92	0.33

Note: The values for population, GDP, and GDP *per capita* were obtained from (World Bank, 2025) on 24 July 2024. PRC and USA refer to the People’s Republic of China and the United States of America, respectively.

Table 2 shows that Italy leads in the number of publications with 105, accounting for approximately 15 percent, followed by the PRC with 77 publications, or about 11 percent. It also indicates that 19 countries have contributed to the advancement of the cobotics field with at least 10 publications each. All Group of Seven (G7) countries—Canada, France, Germany, Italy, Japan, England, and the USA—are active in this research area. Among BRICS countries—Brazil, Russia, India, the PRC, South Africa, Iran, Egypt, Ethiopia, and the United Arab Emirates (UAE)— the PRC is the leading contributor. Also, articles from Thailand, Switzerland, and Egypt have garnered significant attention in the cobot research community, with average citation counts of 112, 52.5, and 39.5, respectively, despite having only 2, 8, and 2 publications. The high average citation rates, combined with the relatively few publications from these countries, highlight the sensitivity of this metric; a small number of highly cited papers can significantly influence the average citation counts when the total number of publications is low.

To more effectively assess a country’s research productivity in a specific area, the number of articles *per capita*, NAC , and the number of articles *per capita* Gross Domestic Product (GDP), NAG , can be used (see Equations 5 and 6) (Amin et al., 2019; Alauddin et al., 2018).

$$NAC=\frac{Totalnumberofarticles}{Population}$$ (5)

$$NAG=\frac{Totalnumberofarticles}{GDPpercapita}$$ (6)

where GDP per capita is given by Equation 7.

$$GDPpercapita=\frac{GDP}{Population}$$ (7)

**FIGURE 4:**
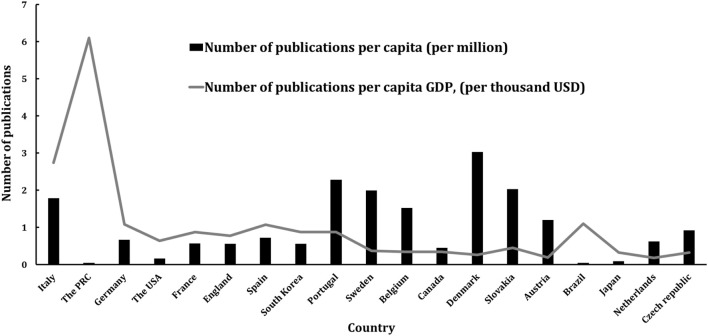
Research efficiency indices of top contributing countries.

The NAC reflects the proportion of a country’s population engaged in research, while the NAG indicates how effectively a country’s financial resources are utilized for research. As shown in Table 2 and Figure 4, Denmark has the highest NAC , with 3.03 articles per million people, followed by Portugal at 2.28 and Slovakia at 2.03 articles per million. In contrast, the PRC and Brazil have the lowest NAC scores, both at 0.05 articles per million. Regarding financial support, the PRC leads with an NAG score of 6.1 articles per thousand USD, followed by Italy at 2.74 articles per thousand USD, and Brazil at 1.1 articles per thousand USD.

#### 3.2.2 Research institutions

**FIGURE 5:**
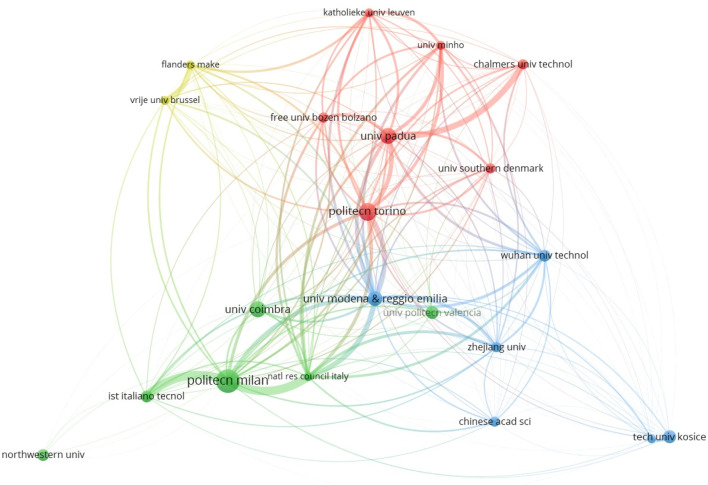
Bibliographic coupling among research institutions with at least six published papers.

An analysis reveals that 570 research institutions have contributed to the 532 retrieved articles advancing the field of cobotics for industry. Among these, 21 institutions have published six or more papers. As shown in Table 3, the most productive institution in cobot research is the *Politecnico di Milano*, with 16 publications. This is followed by the *Politecnico di Torino* with 12 publications, and the University of Padua and the University of Coimbra, each with 11 publications. Among the 21 institutions publishing six or more papers, the University of Modena and Reggio Emilia stands out, having attracted the most attention from scholars based on both the total number of citations and the average number of citations. Notably, 7 of the top 21 institutions are from Italy, highlighting the country’s dominance in this research area. Figure 5 represents the bibliographic coupling among research institutions. Bibliographic coupling occurs when publications from two institutions reference works from a third common institution. Each research institution is represented by a sphere, with the size of the sphere and its label indicating the magnitude of the institution’s contribution. The larger the sphere and label, the greater the contribution. The colors represent clusters of institutions, while the thickness of the arcs between spheres represents the strength of the bibliographic coupling.

**TABLE 3:** Top contributing institutions to the field of cobotics for industry.

Rank	Research institutions	Country	Number of articles	% of articles	Number of citations	Average number of citations
1	Politecnico di Milano	Italy	16	3.01	515	32.19
2	Politecnico di Torino	Italy	12	2.26	319	26.58
3	University of Padua	Italy	11	2.07	273	24.82
4	University of Coimbra	Portugal	11	2.07	295	26.82
5	University of Modena and Reggio Emilia	Italy	10	1.88	701	70.10
6	Universitat Politècnica de València	Spain	9	1.69	121	13.44
7	Technical University of Košice	Slovakia	9	1.69	66	7.33
8	Wuhan University of Technology	PRC	8	1.50	166	20.75
9	Istituto Italiano di Tecnologia	Italy	8	1.50	347	43.38
10	Northwestern University	USA	8	1.50	505	63.13
11	Zhejiang University	PRC	7	1.32	168	24.00
12	Free University of Bozen-Bolzano	Italy	7	1.32	178	25.43
13	University of Southern Denmark	Denmark	7	1.32	228	32.57
14	Chalmers University of Technology	Sweden	7	1.32	52	7.43
15	Chinese Academy of Sciences	PRC	7	1.32	90	12.86
16	National Research Council of Italy	Italy	6	1.13	208	34.67
17	University of Minho	Portugal	6	1.13	107	17.83
18	Katholieke Universiteit Leuven	Belgium	6	1.13	80	13.33
19	Flanders Make	Belgium	6	1.13	104	17.33
20	Vrije Universiteit Brussel	Belgium	6	1.13	151	25.17
21	VSB — Technical University of Ostrava	Czech republic	6	1.13	46	7.67

### 3.3 Potential sources

**FIGURE 6:**
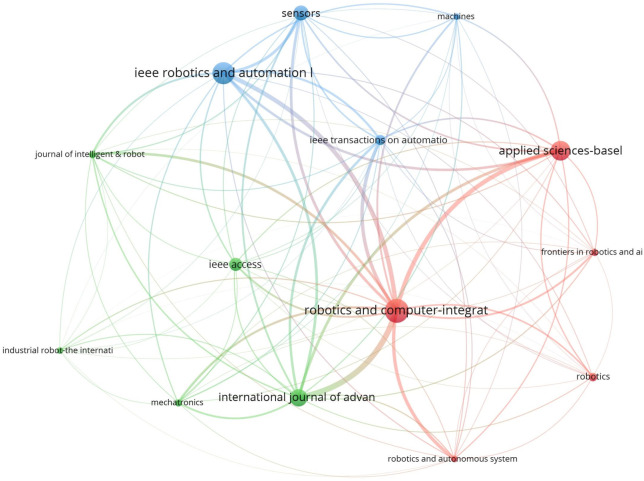
Bibliographic coupling among publication sources with at least 10 papers.

The analysis of publication sources is essential to identify the main journals in a research field, to help researchers find relevant literature and select the right journals for their work. The analysis of 532 selected papers revealed that they came from 156 different journals. Table 4 lists journals that have published more than 10 papers on cobotics for industry. The journal “Robotics and Computer-Integrated Manufacturing” is the leading source, with 35 publications, followed by “IEEE Robotics and Automation Letters” with 33 papers, “Applied Sciences-Basel” with 29 papers, and the “International Journal of Advanced Manufacturing Technology” with 26 papers. Regarding citations, “Robotics and Computer-Integrated Manufacturing” ranks first with 1,602 citations, followed by the “International Journal of Advanced Manufacturing Technology” with 760 citations, and “IEEE Robotics and Automation Letters” with 734 citations. However, papers published in “Mechatronics” attracted the most attention on average, with 62.09 citations per paper, while “Robotics and Computer-Integrated Manufacturing” ranks second with 45.77 average citations. Figure 6 illustrates the bibliographic coupling among various publication sources active in the cobotics with at least 10 papers. The size of each sphere indicates the journal’s relative strength in publishing cobotics papers, the color represents clusters of journals, and the thickness of the arcs between journals indicates the strength of their bibliographic coupling.

**TABLE 4:** Contributions of publication sources to the field of cobotics for industry.

Rank	Publication sources	Publishing model	2023 impact factor	Number of articles	% of articles	Number of citations	Average number of citations
1	Robotics and Computer-Integrated Manufacturing	Hybrid	9.1	35	6.58	1,602	45.77
2	IEEE Robotics and Automation Letters	Hybrid	4.6	33	6.20	734	22.24
3	Applied Sciences-Basel	Open access	2.5	29	5.45	362	12.48
4	International Journal of Advanced Manufacturing Technology	Hybrid	2.9	26	4.89	760	29.23
5	Sensors	Open access	3.4	22	4.14	267	12.14
6	IEEE Access	Open access	3.4	20	3.76	285	14.25
7	IEEE Transactions on Automation Science and Engineering	Hybrid	5.9	15	2.82	562	37.47
8	Robotics	Open access	2.9	13	2.44	133	10.23
9	Frontiers in Robotics and AI	Open access	2.9	11	2.07	71	6.45
10	Journal of Intelligent & Robotic Systems	Open access	3.1	11	2.07	199	18.09
11	Mechatronics	Hybrid	3.1	11	2.07	683	62.09
12	Industrial Robot - The international journal of robotics research and application	Hybrid	1.9	10	1.88	212	21.20
13	Machines	Open access	2.1	10	1.88	77	7.70
14	Robotics and Autonomous Systems	Hybrid	4.3	10	1.88	325	32.50
15 to 156	Other 142 journals	-	-	276	51.88	5,528	20.03

### 3.4 The most productive and influential authors and their cooperation network

Analyzing the number of publications and citations of authors, and mapping co-authorship relationship, allows for the identification of the most productive and influential authors and the most prominent research groups in the field, according to the PRISMA-ScR process applied for literature selection (see Figure 1). This information is invaluable for researchers seeking collaboration and insights from leading experts. The 532 retrieved papers were contributed by 1704 authors, with 26 authors having at least 5 publications, and 26 authors having a minimum of 200 citations (see Table 5). Understanding authors’ contributions to the cobotics research field requires evaluating both the number of articles and citations, and authors are ranked based on these metrics in Table 5. Even though this paper reviewed as many relevant papers as possible, the results are limited to documents retrieved through the PRISMA-ScR process described in Figure 1. For example, some publications may not be indexed in the WoS database, may have been missed due to the chosen search string, may have been excluded based on criteria such as being conference proceedings. These limitations could introduce inaccuracies. Another potential source of bias is the inability to distinguish between authors with identical names. Likewise, authors who publish under different names may not be correctly linked. To address this issue, it is strongly recommended that researchers be assigned a unique, persistent identifier, such as the Open Researcher and Contributor ID (ORCID), upon publishing their first paper. This would help resolve such issues, regardless of how a researcher’s name appears across different publications (van Nunen et al., 2018; Chiu and Ho, 2007).

**TABLE 5:** Contributions of authors to the field of cobotics for industry.

Ranked by number of articles					Ranked by number of citations				
Rank	Author	Number of articles	Number of citations	Average number of citations	Rank	Author	Number of articles	Number of citations	Average number of citations
1	Vidoni, R	8	195	24.38	1	Secchi, C	4	600	150.00
2	Vanderborght, B	8	155	19.38	2	Villani, V	2	549	274.50
3	Neto, P	7	247	35.29	3	Leali, F	2	547	273.50
4	Rocco, P	7	184	26.29	4	Pini, F	2	547	273.50
5	Zanchettin, AM	7	184	26.29	5	Cherubini, A	4	460	115.00
6	Antonelli, D	6	140	23.33	6	Passama, R	3	456	152.00
7	Colim, A	6	107	17.83	7	Colgate, J	5	442	88.40
8	Gracia, L	6	99	16.50	8	Crosnier, A	3	394	131.33
9	Tornero, J	6	99	16.50	9	Fraisse, P	3	394	131.33
10	Palmieri, G	6	63	10.50	10	Lasnier, A	2	376	188.00
11	Colgate, J	5	442	88.40	11	Malik, AA	5	357	71.40
12	Malik, AA	5	357	71.40	12	Peshkin, M	3	339	113.00
13	Vicentini, F	5	215	43.00	13	Gillespie, R	2	290	145.00
14	Safeea, M	5	194	38.80	14	Moore, C	2	269	134.50
15	Faccio, M	5	172	34.40	15	Neto, P	7	247	35.29
16	Gualtieri, L	5	157	31.40	16	Wang, L	4	240	60.00
17	Rauch, E	5	157	31.40	17	Lasota, PA	2	233	116.50
18	Pang, G	5	149	29.80	18	Shah, JA	2	233	116.50
19	Yang, G	5	149	29.80	19	El Zaatari, S	4	225	56.25
20	Wang, Y	5	148	29.60	20	Li, W	4	225	56.25
21	El Makrini, I	5	134	26.80	21	Akella, P	1	220	220.00
22	Solanes, JE	5	94	18.80	22	Wannasuphoprasit, W	1	220	220.00
23	Song, JB	5	88	17.60	23	Vicentini, F	5	215	43.00
24	Bogue, R	5	62	12.40	24	Bilberg, A	3	205	68.33
25	Scoccia, C	5	59	11.80	25	Usman, Z	2	203	101.50
26	Bobovsky, Z	5	43	8.60	26	Marvel, JA	3	200	66.67

Vidoni, R., and Vanderborght, B. lead the ranking in terms of the number of articles, with 8 papers each. Vidoni, a professor at the Free University of Bozen-Bolzano (Bolzano, Italy), specializes in high-performance (energy, vibration, safety, collaborative) automatic machines. Vanderborght, a professor at the Vrije Universiteit Brussel (Brussels, Belgium), focuses on cognitive and physical human-robot interaction, robot-assisted therapy, humanoids, and rehabilitation robotics using variable impedance actuators. In terms of citations, Secchi, C. leads with 600 citations. Secchi is a professor at the University of Modena and Reggio Emilia, known for his work in human-robot collaboration, multi-robot systems, and medical robotics.

**FIGURE 7:**
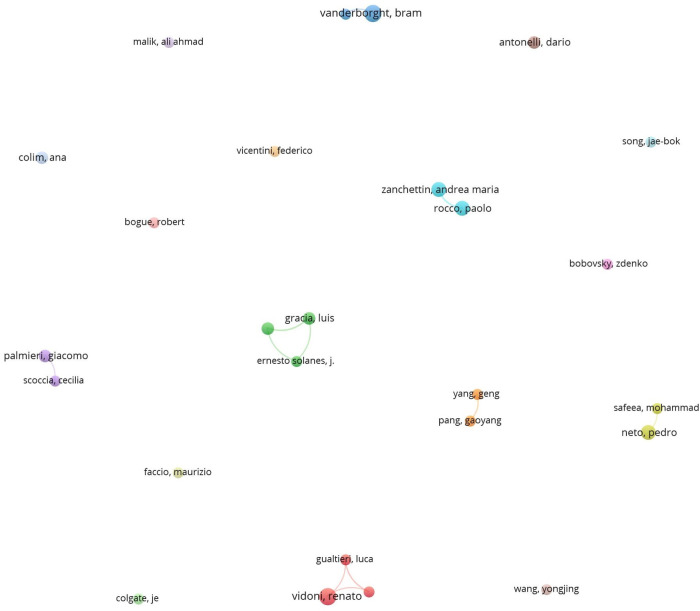
Co-authorship network with at least five publications.

Figure 7 illustrates co-authorship in the cobotics research field for industry, with spheres representing authors, colors indicating clusters of authors, and arcs showing co-authorship strength. It reveals that the international community actively engaged in cobotic research remains limited.

### 3.5 Prominent research topics

**FIGURE 8:**
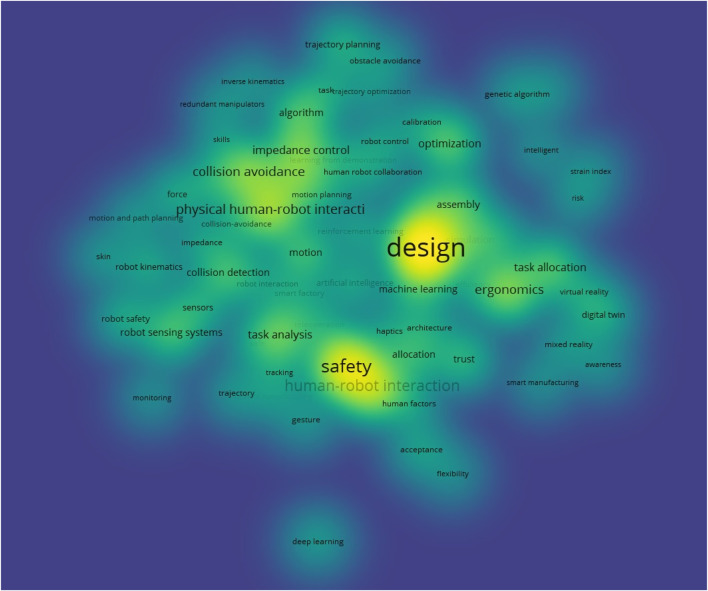
Density of term co-occurrence with a minimum of five occurrences.

Analyzing the frequency of terms in the 532 retrieved papers offers valuable insights into the main research topics within the field of cobotics for industry. To identify the most prominent topics, a terms co-occurrence density map was constructed. Terms appearing in at least five papers were included, while general terms such as “work”, “model”, or “methodology”, as well as cobot-specific terms like “robot”, “collaboration”, “collaborative robot”, or “cobot”, were excluded. This process identified a total of 71 relevant terms, with their co-occurrence density visualized in Figure 8.

## 4 Content analysis–Results and discussions

**FIGURE 9:**
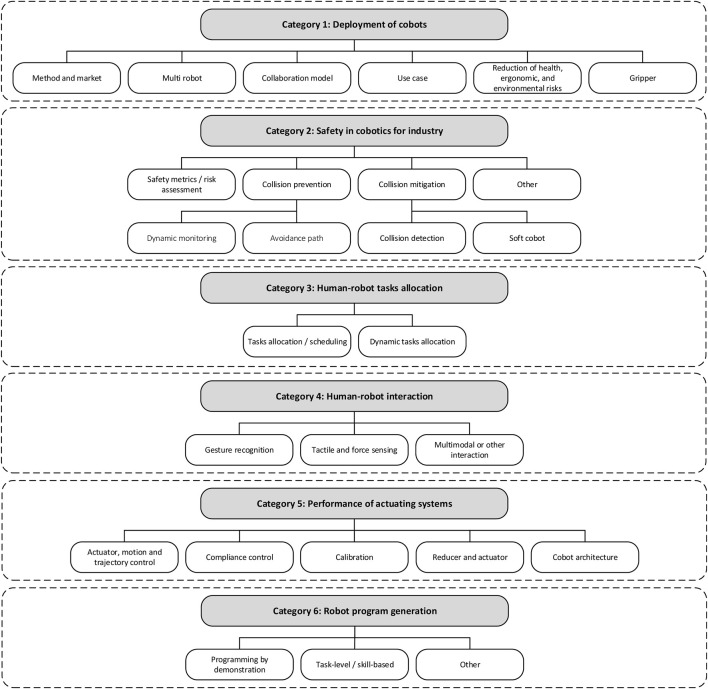
The framework for developing research topics in cobotics for industry.

In this section, we provide the results and discussions of the content review. The content analysis of the paper resulted into a univocal categorization framework, along with a bibliometric analysis for each category. A thorough analysis of the terms on the map (Figure 8) led to creating 25 sub-categories of research topics. Afterwards, those sub-categories have been grouped into six categories, as shown in Figure 9. The categories are: 1) deployment of cobots, 2) safety in cobotics for industry, 3) human-robot tasks allocation, 4) human-robot interaction, 5) performance of actuating systems, and 6) robot program generation. In this section, we provide a high-level overview of existing viewpoints by categorizing and discussing them using the categorization framework. A brief discussion of the research topics (i.e., categories) is presented in the following subsections.

### 4.1 Deployment of cobots

The category “Deployment of cobots” is how to deploy cobots in an industrial organization. Methodologies for deployment are studied to optimize cobots’ integration. Furthermore, comparing traditional robots versus cobots (Faccio et al., 2019), maximizing with other enabling technologies (e.g., virtual reality and digital twins) are some examples of research outputs. For instance, using a digital twin, which is a virtual replica (model) of a real system, can guide and enhance the performance of the actual system. Several researchers put efforts to explore the application of digital twins (Gallala et al., 2022; Lima et al., 2019; Malik and Brem, 2021; Ronzoni et al., 2021), virtual reality (Badia et al., 2022), mixed reality (Ostanin et al., 2021), or a combination of them (Pérez et al., 2020; Wolfartsberger et al., 2019), to address complexities in workspaces where humans and cobots interact with each other.

Growing interests on cobots in commercial markets has been investigated in the literature. These papers presented here explore the market potential and future prospects of cobots. For example, scholars have predicted that the significance of cobots will continue to grow (Bogue, 2022; Bogue, 2016b; Bloss, 2016). Although the cobotic is a relatively new research topic in the industrial robotics, the applications of cobots are rapidly expanding in industrial sectors such as assembly, packaging, and surface treatment. This category also covers the papers that use cases explained how to deploy efficiently cobots for studied applications. Table 6 presents an outline of the deployment of collaborative applications.

**TABLE 6:** Examples of the industrial collaborative-application-related research.

References	Cobot name	Case studies and applications
Vocetka et al. (2020)	YuMi	Assembly
Colim et al. (2021a), Kunic et al. (2021)	UR10e	
Murali et al. (2020), Palomba et al. (2021), Andronas et al. (2022)	UR10	
Scimmi et al. (2021), Antonelli et al. (2021), Realyvásquez-Vargas et al. (2019)	UR3	
Kunic et al. (2021)	UR5e	
Alvarez-de-los-Mozos et al. (2020)	KUKA Lightweight Robot	Disassembly
Li et al. (2020a), Huang et al. (2019), Huang et al. (2020)	KUKA LBR iiwa 14 R800	
Huang et al. (2021)	KUKA LBR iiwa 14 R820	
Mathew et al. (2022)	KUKA KMR iiwa	Material handling
Zaccaria et al. (2021)	UR10e	
Comari et al. (2022)	Lightweight LBR iiwa 14 manipulator	
Hayakawa et al. (2022)	UR3	
Tannous et al. (2020)	Mitsubishi MELFA RV-13FM-D	Welding
Canfield et al. (2021)	AUBO i5	
Ochoa and Cortesao (2022)	Panda/ Franka Emika	Surface treatment
Ubeda et al. (2021)	UR3	
Chiriatti et al. (2022)	UR5e	
Gracia et al. (2019)	Sawyer	
Lakshminarayanan et al. (2021)	KUKA LBR iiwa 7 R800	
O'Shea et al. (2021)	IRB 14000 Yumi	Other (e.g., drilling, construction, mining, measurement, testing, inspection, and maintenance)
Rossi et al. (2020)	ABB IRB 14050 Single-arm YuMi	
Aydin et al. (2021)	LBR iiwa 7 R800, KUKA	
Raviola et al. (2021a), (Riedl et al. (2019)	UR10	
Reinhardt et al. (2020)	Kuka’s iiwa/ABB’s Yumi	
Safeea et al. (2022)	KUKA iiwa 7R800	
Safeea et al. (2022)	KUKA iiwa 14R820	
Kim and Choi (2022)	Niryo One (NIRYO)	
Maithani et al. (2021)	KUKA LWR Robot	
Sultan et al. (2022)	UR3e	
Zhou et al. (2022)	UR5e	
Pollák and Kocisko (2021)	UR5	

Despite increasing cobot-related applications, their adoption remains inadequate in real environments. Therefore, researchers investigate on effective parameters on cobots’ adoption such as trust between operator and cobot. Styles of human-robot symbiosis are modelled or experienced to improve trust and operators’ satisfaction as well as performance (Lambrechts et al., 2021; Zemlyak et al., 2022; Baumgartner et al., 2022; Quintana et al., 2022; Mateus et al., 2019; Andersson et al., 2021; Liu and Cao, 2022; Simoes et al., 2020; Bagheri et al., 2022; Kopp et al., 2022; Maurtua et al., 2017a; Sauer et al., 2021). Clearly enough, successfully deploying a cobot in a system depends on various factors and can have both positive and negative impacts. Therefore, evaluating the cobot’s impacts (e.g., economic, social, environmental impacts) before deployment is crucial. This category can contain works that investigate the impacts of cobots, as well as those that develop models at improving these impacts (Alvarez-de-los-Mozos et al., 2020; Ronzoni et al., 2021; Colim et al., 2021a; Palomba et al., 2021; Realyvásquez-Vargas et al., 2019; Chiriatti et al., 2022; Colim et al., 2021b; Zhang YJ. et al., 2021; Ojstersek et al., 2022; Calvo and Gil, 2022; Navas-Reascos et al., 2022; Javernik et al., 2022; Kim et al., 2021; El Makrini et al., 2022; Liu and Wang, 2020; Gualtieri et al., 2020; Ibáñez et al., 2021).

As discussed above, one of the advantages of implementing cobots is the reduction of health, ergonomic, and environmental risks (which is a social consideration), such as biomechanical overload. Industrial workers face to various ergonomic hazards such as improper postures, repetitive motions, heavy lifting, and vibrations. Several techniques are available to assess ergonomic risk factors, including observational, tool-based, and questionnaire-based methods (Cheraghi et al., 2019). Among these, observational methods like Rapid Upper Limb Assessment (RULA) (McAtamney and Corlett, 1993), Rapid Entire Body Assessment (REBA) (Hignett and McAtamney, 2000), Posture, Activity, Tools and Handling (PATH) (Buchholz et al., 1996), and the Ovako Working Posture Analysing System (OWAS) (Karhu et al., 1977) are widely used. These methods have been extensively applied for ergonomic evaluation of collaborative systems, including RULA (Colim et al., 2021a; Palomba et al., 2021; Chiriatti et al., 2022; Colim et al., 2021b; Navas-Reascos et al., 2022; Gualtieri et al., 2020; Ibáñez et al., 2021; Colim et al., 2021c), the Strain Index (SI) (Colim et al., 2021b; Zhang YJ. et al., 2021), NIOSH indices (Ronzoni et al., 2021), REBA (El Makrini et al., 2019), KIM-MHO (Colim et al., 2021b; Colim et al., 2020), EAWS (Maurice et al., 2019), JSI (Navas-Reascos et al., 2022), and OCRA (Ronzoni et al., 2021; Gualtieri et al., 2020). Despite their popularity, conventional techniques for evaluating ergonomic conditions in cobot-related workspaces have limitations: they may not cover all types of activities in a collaborative environment, and they are incapable of addressing dynamic phenomena, such as fast motions. Consequently, it is more accurate to employ a technique specifically designed for assessing ergonomic risks of collaborative jobs. To address this need, researchers can be attracted to propose methods specifically developed for evaluating ergonomic risks associated with collaborative activities (Maurice et al., 2017).

Grippers are the most widely adopted end-effectors in collaborative industrial applications. They are essential for tasks requiring precision and versatility like human-hand capabilities, making them an interesting research topic in the field of cobotics. Grippers must be designed to be cost-effective and capable of handling a wide range of objects that vary in size, shape, and stiffness. In addition, they should ensure operator safety by limiting contact forces and avoiding sharp edges and pinch points. This sub-category has been attracted a lot of attentions of the cobotic researchers (Bogue, 2016a; Birglen, 2019; Iqbal et al., 2021; Anwar et al., 2019).

### 4.2 Safety in cobotics for industry

This category represents how to guarantee the safety of the operator while optimizing the performance of the cobot. It is mainly dedicated to the risk of an unwanted collision between the cobot and the operator. This category comprises papers discussing standards for cobot safety and how to meet them. According to those papers, the main safety standards related to robotic are Section IV of Chapter 4 of OSHA Technical Manual (OSHA, 2017), ANSI/RIA R15.06 (Association, 2012), ISO 10218-1 (ISO, 2011a), and ISO 10218-2 (ISO, 2011b). Among these standards, the most relevant standard addressing cobots in addition to conventional industrial robots is ISO 10218, Parts 1 and 2. While the papers analyzed regarding the safety category used the 2011 version of ISO 10218, a recent version of that standard was issued in 2025, i.e., after the publication of those papers. The 2011 version of part 2 introduced the concept of “collaborative robots”, which is no longer used in its 2025 version (ISO, 2025a; ISO, 2025b). The latter focuses more on the collaborative aspect of the robot application. However, to design a safe collaborative workspace, a technical specification ISO/TS 15066 (ISO, 2016), that complements the former version of ISO 10218 was needed. Now, most of that TS is available and updated in the 2025 version of ISO 10218.

The ISO 10218:2011 and ISO/TS 15066:2016 cited in the papers introduced four safety methods for a collaborative application: (i) safety-rated monitored stop (SRMS), (ii) hand guiding (HG), (iii) SSM, and (iv) PFL. The 2025 version of ISO 10218 now only considers methods (ii) to (iv) for collaborative applications. Even though SRMS is not in the 2025 version, the following paragraph describes succinctly each of the four methods for better understanding of their meaning.

In the SRMS method, both the operator and cobot could not operate simultaneously within the shared space. To avoid an unwanted collision, the cobot would have been stopped when it detects an operator inside the shared space. In the HG method, the operator directly teaches the cobot by physically moving it. In the SSM method, the cobot’s speed adjusts based on the separation distance between the operator and the cobot. The PFL method is prone to unintentional collisions. It limits the quasi-static contact (i.e., contact between an operator and part of a robot system, where the operator body part can be clamped between a moving part of a robot system and another fixed or moving part of the robot cell) and transient contact (i.e., contact between an operator and part of a robot system, where the operator body part is not clamped and can recoil or retract from the moving part of the robot system) forces below specific thresholds to mitigate human injuries. Achieving that involves active safety measures like compliance control and passive safety measures like edge smoothing during the cobot’s design stage. Based on ISO 10218-2:2025, hazard identification and risk analysis is needed to identify the hazards and assess the risks of both the cobot system and the collaborative environment and then select the proper safety measures (ISO, 2011a).

The developed methodologies in the literature related to the risk of collision between a cobot and an operator can be classified into two main groups: (i) pre-collision (collision prevention), and (ii) post-collision (collision mitigation). The pre-collision approach aims to ensure the safety of operators by detecting potential collisions between the cobot and the operator. This detection is achieved through safety sensor systems such as proximity and visual sensors, or by predicting unintended collisions using stochastic or machine learning methods. Once a potential collision is identified, preventive measures are implemented to avoid it (Li et al., 2023; Gualtieri et al., 2021a; Zorman et al., 2023). In the context of pre-collision strategies, the former SRMS, as well as the actual HG, and SSM safety methods played a key role in the papers analyzed.

Dynamic monitoring allows to know where are located the robot and the operator in unstructured and time-varying environment using advanced sensing technologies. Various approaches have been developed, including the use of virtual reality, Internet of Things (IoT), and sensing technologies to monitor humans in robot workspaces, aiming to prevent collisions (Tsuji and Kohama, 2019; Farsoni et al., 2022; Guerra et al., 2016; Gradolewski et al., 2020; Geiger and Waldschmidt, 2019; Ferraguti et al., 2020; Costanzo et al., 2022; Bin Islam et al., 2019; Barbosa et al., 2022; Safeea and Neto, 2019; Benli et al., 2019; Kianoush et al., 2021). Effective collision avoidance and path optimization are crucial for maintaining safety and productivity in collaborative applications. Therefore, several studies have focused on pre-collision algorithms and dynamic path planning to minimize collision risks and improve cycle times (Scimmi et al., 2021; Kot et al., 2022; Scalera et al., 2022; Chiriatti et al., 2021; Zanchettin et al., 2022; Chan and Tsai, 2020). In addition, the visibility of the robot to the human operator can significantly impact both the operator’s comfort and the probability of collisions. This critical factor has been examined in several research papers with the aim of enhancing the safety of collaborative application (Tarbouriech and Suleiman, 2020; Dufour et al., 2020; Najmaei et al., 2010). Readers interested in further exploration of collision avoidance can find successful efforts in (Simas et al., 2022; Scoccia et al., 2021; Safeea et al., 2019; Elguea-Aguinaco et al., 2022).

On the contrary, the post-collision approach focuses on operator safety through the detection of unintended collisions and then mitigation of energy transfer during these collisions that can lead to human injuries or even fatalities (Li et al., 2023; Gualtieri et al., 2021a; Zorman et al., 2023). In the context of post-collision strategies, the PFL method is employed. As mentioned above, although the PFL method allows unintentional collisions, it is required to include contact force measurements to validate safety (Zimmermann et al., 2022). Therefore, techniques (such as statistical models) to determine contact force thresholds for using in PFL method has been proposed (Behrens et al., 2022). Additionally, several testing procedures for validating safety in PFL method have been developed (DGUV, 2017; ANSI, 2018), and the reliability of such tests have been examined (Scibilia et al., 2021).

Soft cobots (i.e., cobots that can deform and yield in a collision) are often equipped with a protective covering, known as “skin”, to offer both active protection (detecting collisions and initiating cobot reactions) and passive protection (providing a cushioning effect to mitigate the consequences of collisions). Therefore, developing cobot skins take a great apportion of studies in this category (Runge and Raatz, 2017; Ye et al., 2022; Svarny et al., 2022; Pang et al., 2021; Nguyen et al., 2021; Ge et al., 2022; Ye ZQ. et al., 2020; O'Neill et al., 2018; Tsuji and Kohama, 2020; Heng et al., 2021). Furthermore, to reduce cobot production costs, sensor-less collision detection methods (e.g., neural network-based approaches) have gained attention (Kwon et al., 2021; Lee and Song, 2016; Heo et al., 2019; Sharkawy et al., 2020a; Ma et al., 2020; Czubenko and Kowalczuk, 2021; Le and Kang, 2022; Yen et al., 2019; Kim, 2022). Many other studies have contributed to the field of collision detection, including (Heo et al., 2019; Abu Al-Haija and Al-Saraireh, 2022; Amin et al., 2020; Huang et al., 2022; Lee et al., 2019; Li W. et al., 2020; Long et al., 2022; Lu SN. et al., 2022; Nguyen and Case, 2022; Park et al., 2022; Ren et al., 2018; Rodrigues et al., 2023; Shin et al., 2019; Strazdas et al., 2021; Xiao et al., 2018; Yun A. et al., 2022; Zhang ZJ. et al., 2021), providing valuable information for researchers. To ensure the operator’s safety in a collaborative workspace, in addition to measuring and controlling the interaction force, other parameters like its direction, the point of application, and the time required to measure these parameters are also important and can form the research objective of a study (Popov et al., 2021).

Several techniques have been developed for identifying and evaluating system hazards, such as Failure Mode and Effect Analysis (FMEA), Job Safety Analysis (JSA), and Fault tree analysis (FTA), as well as for assessing human reliability, including Human Error Assessment and Reduction Technique (HEART) and Cognitive Reliability and Error Analysis Method (CREAM). These generic techniques can be applied to cobotics. However, due to the unique challenges inherent in cobotic applications, some researchers have worked on developing safety metrics and risk assessment tools specifically for these systems (Askarpour et al., 2019; Marvel et al., 2015; Pantano et al., 2022; Vemula et al., 2018; Vicentini et al., 2020). A safety metric or risk assessment tool for cobotics should be able to consider various parameters, such as the uncertainty associated with human operator behaviors, the potential severity of physical contact between the cobot and the operator, and changes within the system. Last but not least, one of the key factors that can impact safe collaboration in cobotics is the threat of cyber-attacks. Defense strategies to protect workers against cyber-attacks have been also investigated (Khalid et al., 2018).

### 4.3 Human-robot tasks allocation

*“Human-robot tasks allocation”* aims to distribute the tasks between humans and cobots. This involves determining the sequence of tasks and deciding which agent (cobot or human) should perform each task. With shorter product life cycles and high-mix production, the job splitting between the agents is becoming increasingly important. Research in this field has focused on optimizing task allocation to enhance efficiency, ergonomics, and economic factors. Optimization models aim to minimize makespan and production costs while considering ergonomic risks and worker wellbeing (El Makrini et al., 2019; Yu et al., 2020; Wang et al., 2018; Nourmohammadi et al., 2022; Mura and Dini, 2022; Liu et al., 2024b; Liau and Ryu, 2022a; Gjeldum et al., 2022; Cai et al., 2023; Ferreira et al., 2021; Kinast et al., 2022; Almasarwah et al., 2022; Stecke and Mokhtarzadeh, 2022; Mura and Dini, 2019; Li ZX. et al., 2021; Weckenborg et al., 2022; Pearce et al., 2018; Boschetti et al., 2021a).

As revealed in the above-reviewed papers, optimal human-robot task allocation involves balancing objectives like minimizing cycle time, costs, and ergonomic risks. Selecting the best approach is challenging due to conflicting requirements. For instance, minimizing production costs by assigning more tasks to cheaper work cells may delay deliveries, while minimizing makespan may increase costs. Additionally, relying solely on these objectives may lead to assigning high-risk tasks to human workers. Thus, the collaborative applications designers should comprehensively consider economic, social, and environmental aspects to ensure sustainable task allocation in collaborative applications.

These approaches are powerful in a deterministic collaborative environment, where both the operator and the cobot must adhere to a pre-planned task sequence. However, in real collaborative task-allocation problems, various uncertain parameters, especially those related to the operator, should be considered. For example, the operator may deviate from the task sequence, either by mistake or based on personal preference. Such deviations may not yield the predefined optimal solution but could have a negligible impact on the objectives of the system. In such scenarios, the process may halt if the cobot cannot adapt to new conditions or human actions. Therefore, a cobot should be capable of finding alternative solutions while still maintaining the original objectives. To address these uncertainties, dynamic task allocation models have been developed (Antonelli et al., 2021; Pupa et al., 2021; Petzoldt et al., 2022; Chacón et al., 2021; Messeri et al., 2022; Casalino et al., 2021; Antonelli and Bruno, 2019; Bruno and Antonelli, 2018; Pupa et al., 2022).

### 4.4 Human-robot interaction

“Human*-robot interaction”* gathers research on the way that the human and the robot interact themselves (co-working) in order to perform their allocated industrial actions. In the collaborative application, the operator often defines the objective of collaborative work and the cobot assists the operator to achieve this objective. The cobot must therefore be able to learn from the operator and estimate his intentions. Various input interaction modes are explored to instruct the cobot about the state of humans or their intentions such as gesture visual recognition and force sensing.

Gesture (e.g., hand, body, and head) visual recognition is one of the most powerful communication mode which can be developed by techniques such as wearable sensors, inertial measurement unit (IMU) sensors surface electromyography (EMG) signals (Mukherjee et al., 2022; Papanagiotou et al., 2021; El Aswad et al., 2021; Coupeté et al., 2019; Mendes, 2022; Cornak et al., 2022; Tang et al., 2015; Shukla et al., 2018; Lee et al., 2022). Besides vision-based systems, wearable systems capable of measuring joint rotation can easily capture an operator’s gestures. However, a primary limitation of such systems is their potential interference with the operator’s work. In addressing this challenge, wireless wearable system designed to determine the orientation of the operator’s upper body parts has been developed (Skulj et al., 2021). Tactile sensing allows cobots to detect and interpret pressure and force exerted by human operators (Olivares-Alarcos et al., 2019; Bauer et al., 2008). Cobotics leveraging tactile sensors can dynamically determine human grasp positions and intentions being able to ability to adapt to human actions and improve overall interaction efficiency (Li TJ. et al., 2021; Ansari et al., 2020). Overall, numerous papers in this category focus on designing and developing new sensors to improve cobotic systems and increasing their cost-effectiveness (Zaid et al., 2022; Long et al., 2021; Fu and Cai, 2022; Castano-Cano et al., 2022). In human-robot interactions, relying solely on mechanical force-torque sensors to measure forces can sometimes lead to the unintentional measurement of additional forces arising from the robot’s contact with an unpredictable environment. This can potentially introduce inconsistencies in human-robot interactions (Ajoudani et al., 2018). As an alternative approach, bio-signals, like EMG signals, can be used to directly measure the forces exerted by the human operator (Bednarczyk et al., 2022).

In a collaborative application, communication methods extend beyond gesture recognition and force sensing to include natural speech, gaze, graphical signage, and physiological signals (e.g., electroencephalographic (EEG)) (Dmytriyev et al., 2022; Eimontaite et al., 2022; Eimontaite et al., 2019). Natural speech and gaze are also crucial for effective human-robot interaction. Speech control systems allow operators to command multiple robots in different languages (Hofer and Strohmeier, 2019). Also, using EEG signals, emotional states (e.g., fearful) of operators can be assessed in a cobotic system (Buerkle et al., 2021; Borboni et al., 2022; Eyam et al., 2021). In addition, to enhance communication reliability, some researchers have adopted multimodal interaction modes to provide complementary or redundant input options (Tsuji and Kohama, 2022).

### 4.5 Performance of actuating systems

“Performance of actuating systems” is how to improve the actuating system of a cobot through its logical or physical equipment. The control of trajectory and motion is addressed in this category. To achieve high efficiency, precision, and safety in cobotics, numerous researchers have made efforts to develop trajectory and motion planning methods. These methods aim to balance performance with psychological and physical safety of the operator (Zhao et al., 2022; Ye L. et al., 2020; Wittmann and Rixen, 2022; Vysocky et al., 2020a; Sidobre and Desornneaux, 2019; Magyar et al., 2019; Li et al., 2019; Krämer and Bertram, 2022; Kraemer et al., 2020; Chen et al., 2018; Liu et al., 2022a; Rojas et al., 2019; Rojas et al., 2020; Zhu et al., 2022; Palleschi et al., 2021).

Force control was originally developed for applications where the robot’s end-effector comes into direct physical contact with its environment, often involving tasks like surface treatments, such as polishing and grinding (Ochoa and Cortesao, 2022; Ubeda et al., 2021; Gracia et al., 2019; Lakshminarayanan et al., 2021; Perez-Vidal et al., 2019). These scenarios require precise force management to achieve the desired quality of work. Due to the safety concerns, force control and its variants, such as impedance control and admittance control, become increasingly significant in a collaborative system. Impedance control involves computing the robot’s resistance to motion when external forces are applied, while admittance (i.e., the inverse of the impedance) control focuses on adjusting the robot’s trajectory in response to external forces (Siciliano and Villani, 1999; Khan et al., 2014). Impedance/admittance control is commonly employed in cobotics for physical interactions among humans, cobots, and the environment (Aydin et al., 2021; Li et al., 2020c; Kim and Yang, 2021; Dou et al., 2022). Compliance (the opposite of stiffness), i.e., the ability to yield or adapt to external forces, can be achieved through (i) active compliance, and (ii) passive compliance. Active compliance is a software-based approach, allowing the robot to dynamically respond to external forces. Passive compliance, on the other hand, is designed into the robot’s mechanical structure, allowing it to naturally respond to external forces. Both approaches have been extensively investigated in the literature (Huang et al., 2019; Huang et al., 2020; Huang et al., 2021; Zhang SL. et al., 2021; Zeng F. et al., 2019).

The ability to vary stiffness is crucial in cobotics. High stiffness is employed for regular operational routines, while reduced stiffness can minor contact forces or even be a detector of collisions (Ayoubi et al., 2019; Ayoubi et al., 2020; Liu YW. et al., 2021). In addition, variable stiffness enables the cobot to perform diverse tasks (Ren et al., 2019a). Clearly enough, accuracy is a critical performance characteristic of a cobotic system, particularly in applications that demand precise positioning, manipulation, or sensing. Calibration is the process that guarantees the cobotic system achieves the desired accuracy. Research in this area focuses on improving metrology system and calibration techniques (Pagani et al., 2021; Yun HT. et al., 2022; Kim et al., 2022; Pollák et al., 2020; Kolyubin et al., 2019).

In the literature, some concepts of novel architecture of cobots are explored, such as modular components between cobots, two armed cobot, or extra components to reduce inertia. For instance, the integration of two cobotic arms significantly enhances the potential of cobotics for industrial applications, allowing for tasks such as handling bulky and heavy objects (Liu LY. et al., 2021; Cherubini et al., 2019). In addition, as discussed above, one of the most significant advantages of cobots over traditional industrial robots is their adaptability to a wide range of tasks. However, achieving this flexibility requires more than just reprogramming; the hardware components also need to be reconfigured (Romiti et al., 2022; Smrcek et al., 2022; Schou and Madsen, 2017; Wojtynek et al., 2019).

### 4.6 Robot program generation

*“Robot program generation”* is how to generate the program for the cobot, by benefiting of the collaboration with humans. It is useful to enable a fast and easy-to-attain cobot task reconfiguration, even for inexperienced operators. Programming by demonstration develops a manual physical guiding of the cobot by its end-effector. System records waypoints, force inside joints or gripper. This method has been widely applied in the literature (Canfield et al., 2021; Ochoa and Cortesao, 2022; Wang et al., 2019a; Wang LK. et al., 2022; Wang LK. et al., 2023; Tadese et al., 2021; Svejda et al., 2022; Stenmark et al., 2018; Rozo et al., 2016; Müller et al., 2020; Iturrate et al., 2021; De Winter et al., 2019; Carfi et al., 2020; Al-Yacoub et al., 2021a; Ajaykumar et al., 2021; Wang et al., 2021; Soares et al., 2021; El et al., 2022; Halim et al., 2022; Safeea and Neto, 2022). While “programming by demonstration” requires a lower level of knowledge from the operator, a cobot’s functionality is typically limited to replaying recorded actions. To overcome this limitation, teaching the cobot from visual input through demonstrations has been developed in the literature (De Coninck et al., 2020).

Skill-based programming reaches the same objective of quick and easy programming, enabling cobots to handle a wide range of tasks. In skill-based programming, robots follow a hierarchy that involves lower-level entities called “robot skills”. These skills represent specific actions that the robot is capable of executing. A task (e.g., removing a specific object from a table) is a sequence of the skills (e.g., pick up object) along with specified parameters (e.g., the object to pick up). Skills are pre-defined by the robotics programmer. The sequence of skills and their associated parameters, required to complete a task, is explicitly programmed by non-expert robot operators in the factory (Schou et al., 2018; Pedersen et al., 2016). Skill-based programming has gained the attention of several research studies (Giberti et al., 2022; Polverini et al., 2019; Herrero et al., 2017).

### 4.7 Bibliometric analysis of research topics

**FIGURE 10:**
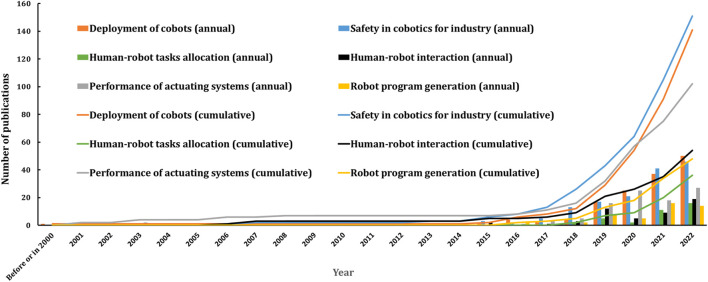
Evolution of research topics over time.

Each of the 532 retrieved papers was classified according to its main research objective. Figure 10 displays both the annual and cumulative number of publications for each research topic. The trends observed conclude that the exponential growth previously identified in Figure 2 is consistent across all research topics. It is evident that publications across all research topics have become more regular since 2016. Also, Table 7 provides the number of papers for each research topic, along with citation analysis and details on the most productive authors, institutions, countries, and leading journals. The most popular research topics are “safety in cobotics for industry” with 151 papers, followed by “deployment of cobots” with 141 papers and “performance of actuating systems” with 102 papers.

**TABLE 7:** Bibliometric analysis of research topics.

Research topic categories	Research topic sub-categories	Reference	Number of articles	Number of citations	Average number of citations	Most productive authors	Most productive institutions	Most productive countries	Leading journals
Deployment of cobots	Method and market	Faccio et al. (2019), Pérez et al. (2020), Bogue (2022), Bogue (2016b), Bloss (2016), Andersson et al. (2021), Javernik et al. (2022), Zhang et al. (2022a), Vaher et al. (2021), Tamas and Murar (2019), Stadnicka and Antonelli (2019), Schlette et al. (2020), Rega et al. (2021a), Pinto et al. (2020), Miyake and Kondo (2022), Mitrea and Tamas (2018), Malik et al. (2020), Havard et al. (2019), Gervasi et al. (2020), Gauss et al. (2022), Fournier et al. (2022), Fager et al. (2021), da Silva et al. (2022), Cohen et al. (2022), Chen et al. (2022), Bounouar et al. (2022), Boschetti et al. (2021b)	27	699	25.89	Colim, A (6 publications)	University of Padua (8 publications)	Italy (27 publications)	Robotics and Computer-Integrated Manufacturing & Applied Sciences-Basel (10 publications)
	Multi robot	Ostanin et al. (2021), Tarbouriech et al. (2022), Savazzi et al. (2021), Liang et al. (2019), Fischer et al. (2019), Dehio et al. (2022), Bharti and McGibney (2022)	7	143	20.43				
	Collaboration model	Gallala et al. (2022), Malik and Brem (2021), Badia et al. (2022), Lambrechts et al. (2021), Zemlyak et al. (2022), Baumgartner et al. (2022), Quintana et al. (2022), Liu and Cao (2022), Simoes et al. (2020), Bagheri et al. (2022), Kopp et al. (2022), Maurtua et al. (2017a), Sauer et al. (2021), Ojstersek et al. (2022), Calvo and Gil (2022), Zhang et al. (2022b), Wang et al. (2022b), Verma and Singh (2024), Tao et al. (2021), Schulz et al. (2018), Savkovic et al. (2022), Rossato et al. (2021), Raziei and Moghaddam (2021), Prati et al. (2021), Pizon et al. (2022), Papetti et al. (2023), Onnasch et al. (2022), Oliff et al. (2020), Meissner et al. (2020), Mangat et al. (2021), Malik and Bilberg (2019a), Lee et al. (2020), Inoue et al. (2021), Hostettler et al. (2022), Hopko and Mehta (2024), Fager et al. (2020), Eberle et al. (2020), Dzedzickis et al. (2022), Cencen et al. (2018), Adriaensen et al. (2022)	40	735	18.37				
	Use case	Lima et al. (2019), Wolfartsberger et al. (2019), Vocetka et al. (2020), Kunic et al. (2021), Murali et al. (2020), Palomba et al. (2021), Andronas et al. (2022), Li et al. (2020a), Mathew et al. (2022), Zaccaria et al. (2021), Comari et al. (2022), Hayakawa et al. (2022), Tannous et al. (2020), Chiriatti et al. (2022), Gracia et al. (2019), Lakshminarayanan et al. (2021), O'Shea et al. (2021), Rossi et al. (2020), Aydin et al. (2021), Raviola et al. (2021a), Riedl et al. (2019), Reinhardt et al. (2020), Safeea et al. (2022), Kim and Choi (2022), Maithani et al. (2021), Sultan et al. (2022), Zhou et al. (2022), Pollák and Kocisko (2021), Navas-Reascos et al. (2022), Gualtieri et al. (2020), Ibáñez et al. (2021), Perez-Vidal et al. (2019), Vagas and Galajdova (2022), Pop et al. (2022), Peshkin and Colgate (1999), Perez-Vidal et al. (2018), Malik et al. (2021), Lins et al. (2015), Liau and Ryu (2022b), Gajsek et al. (2020), D'Souza et al. (2020), Cherubini et al. (2016), Arrais et al. (2021), Alpízar-Cambronero (2020)	44	1,032	23.45				
	Reduction of health, ergonomic, and environmental risks	Alvarez-de-los-Mozos et al. (2020), Cardoso et al. (2021), Ronzoni et al. (2021), Colim et al. (2021a), Realyvásquez-Vargas et al. (2019), Colim et al. (2021b), Zhang et al. (2021a), Kim et al. (2021), El Makrini et al. (2022), Liu and Wang (2020), Colim et al. (2021c), Colim et al. (2020), Maurice et al. (2019), Maurice et al. (2017), Pollak et al. (2020), Paliga (2022), Gualtieri et al. (2022a), Borges et al. (2021), Bogataj et al. (2019)	19	597	31.42				
	Gripper	Bogue (2016a), Birglen (2019), Iqbal et al. (2021), Anwar et al. (2019)	4	54	13.5				
	Total		141	3,260	23.12				
Safety in cobotics for industry	Safety metrics/ risk assessment	Bogue (2017), Askarpour et al. (2019), Marvel et al. (2015), Pantano et al. (2022), Vicentini et al. (2020), Weistroffer et al. (2022), Valente et al. (2022), Vagas and Galajdova (2021), Rojas and Vidoni (2021), Rega et al. (2021b), Pauliková et al. (2021), Park et al. (2019), Lippi and Marino (2021), Langlois et al. (2021), Kopp et al. (2021), Kim et al. (2020), Gleirscher et al. (2022), Gideoni et al. (2022), Fraga-Lamas et al. (2022), Cui et al. (2021), Caruana and Francalanza (2021), Callegari et al. (2022), Alhaji et al. (2021), Alhaddad et al. (2019)	24	402	16.75	Vicentini, F & Pang, G & Yang, G (5 publications)	Politecnico di Milano (7 publications)	Italy (32 publications)	Robotics and Computer-Integrated Manufacturing & IEEE Robotics and Automation Letters (13 publications)
	Dynamic monitoring	Tsuji and Kohama (2019), Farsoni et al. (2022), Guerra et al. (2016), Gradolewski et al. (2020), Geiger and Waldschmidt (2019), Ferraguti et al. (2020), Costanzo et al. (2022), Bin Islam et al. (2019), Barbosa et al. (2022), Safeea and Neto (2019), Benli et al. (2019), Kianoush et al. (2021), Wang (2015), Unhelkar et al. (2018), Polverini et al. (2017), Marvel and Norcross (2017), Long et al. (2018), Lasota and Shah (2015), Aliev and Antonelli (2021)	19	710	37.37				
	Avoidance path	Scimmi et al. (2021), Kot et al. (2022), Scalera et al. (2022), Chiriatti et al. (2021), Zanchettin et al. (2022), Chan and Tsai (2020), Tarbouriech and Suleiman (2020), Simas and Di Gregorio (2022), Scoccia et al. (2021), Safeea et al. (2019), Elguea-Aguinaco et al. (2022), Weitschat and Aschemann (2018), Teso-Fz-Betoño et al. (2019), Merchán-Cruz and Morris (2006), Kanazawa et al. (2019)	15	295	19.67				
	Collision detection	Kwon et al. (2021), Lee and Song (2016), Heo et al. (2019), Sharkawy et al. (2020a), Ma et al. (2020), Czubenko and Kowalczuk (2021), Le and Kang (2022), Yen et al., 2019; Kim (2022), Abu Al-Haija and Al-Saraireh (2022), Amin et al. (2020), Huang et al. (2022), Lee et al. (2019), Li et al. (2020b), Long et al. (2022), Lu et al. (2022b), Nguyen and Case (2022), Park et al. (2022), Ren et al. (2018), Rodrigues et al. (2023), Shin et al. (2019), Strazdas et al. (2021), Xiao et al. (2018), Yun et al. (2022a), Zhang et al. (2021b), Popov et al. (2021), Zhang and Hong (2019), Wahrburg et al. (2018), Sharkawy et al. (2020b), Park et al. (2021a), Nascimento et al. (2021), Mayyas et al. (2020), Kot et al. (2021)	33	693	21				
	Soft cobot	Zimmermann et al. (2022), Behrens et al. (2022), Scibilia et al. (2021), Runge and Raatz (2017), Ye et al. (2022), Svarny et al. (2022), Pang et al. (2021), Nguyen et al. (2021), Ge et al. (2022), Ye et al. (2020a), O'Neill et al. (2018), Tsuji and Kohama (2020), Heng et al. (2021), Zeng and Bone (2013), Virgala et al. (2021), Raiola et al. (2018), Pang et al. (2018)	17	382	22.47				
	Other	Bi et al. (2022), Bi et al. (2021), Broum and Simon (2020), Valori et al. (2021), Vicentini (2020), Grushko et al. (2021), Mukherjee et al. (2022), Berx et al. (2022a), Vicentini (2021), Villani et al. (2018), Proia et al. (2022), Khalid et al. (2018), Buerkle et al. (2021), Zhang et al. (2022c), Zacharaki et al. (2021), Weiss et al. (2021), Vitolo et al. (2022), Sorell (2022), Solanes et al. (2018a), Saito and Ikeda (2007), Saenz et al. (2020), Qi et al. (2022), Mateus et al. (2020), Lucci et al. (2020), Liu et al. (2022b), Islam and Lughmani (2022), Hopko et al. (2021), Hanna et al. (2022), Guidi et al. (2021), Gualtieri et al. (2022b), Gandarias et al. (2020), Farsoni et al. (2019), Douthwaite et al. (2021), Dong et al. (2022), Dahl et al. (2022a), Dahl et al. (2021), Chromjakova et al. (2021), Carmichael et al. (2017), Berx et al. (2022b), Berger et al. (2020), Bdiwi et al. (2022), Ayoubi et al. (2018), Askarpour et al. (2021)	43	1,512	35.16				
	Total		151	3,994	26.45				
Human-robot tasks allocation	Tasks allocation/ scheduling	Mateus et al. (2019), El Makrini et al. (2019), Yu et al. (2020), Nourmohammadi et al. (2022), Mura and Dini (2022), Liu et al. (2024b), Liau and Ryu (2022a), Gjeldum et al. (2022), Cai et al. (2023), Ferreira et al. (2021), Kinast et al. (2022), Almasarwah et al. (2022), Stecke and Mokhtarzadeh (2022), Mura and Dini (2019), Li et al. (2021b), Weckenborg et al. (2022), Pearce et al. (2018), Boschetti et al. (2021a), Malik and Bilberg (2019b), Husing et al. (2021), Gualtieri et al. (2021b), Faccio et al. (2020), Cramer et al. (2021), Conti et al. (2022), Cacace et al. (2023), Alessio et al. (2022), Aleotti et al. (2021)	27	709	26.26	Antonelli, D (4 publications)	Politecnico di Torino (4 publications)	Italy (15 publications)	International Journal of Advanced Manufacturing Technology (8 publications)
	Dynamic tasks allocation	Antonelli et al. (2021), Pupa et al. (2021), Petzoldt et al. (2022), Chacón et al. (2021), Messeri et al. (2022), Casalino et al. (2021), Antonelli and Bruno (2019), Bruno and Antonelli (2018), Pupa et al. (2022)	9	198	22				
	Total		36	907	25.19				
Human-robot interaction	Gesture recognition	Papanagiotou et al. (2021), El Aswad et al. (2021), Coupeté et al. (2019), Mendes (2022), Cornak et al. (2022), Tang et al. (2015), Shukla et al. (2018), Lee et al. (2022), Skulj et al. (2021), Zhang et al. (2021d), Wang et al. (2019b), Vysocky et al. (2020b), Simao et al. (2019a), Simao et al. (2019b), Neto et al. (2019), Lemasurier et al. (2021), Digo et al. (2020), Arntz et al. (2022)	18	331	18.39	Neto, P & Gracia, L & Tornero, J (3 publications)	University of Coimbra & Universitat Politècnica de València (3 publications)	USA & France & England (9 publications)	Sensors (4 publications)
	Tactile and force sensing	Olivares-Alarcos et al. (2019), Li et al. (2021c), Ansari et al. (2020), Zaid et al. (2022), Long et al. (2021), Castano-Cano et al. (2022), Bednarczyk et al. (2022), Watson et al. (2020), Solanes et al. (2018b), Singh et al. (2020), Popov et al. (2022), Leonori et al. (2022), Gracia et al. (2018), Girbés-Juan et al. (2022), Faulring et al. (2007), Faulring et al. (2006), Courreges et al. (2019), Boy et al. (2007)	18	198	11				
	Multimodal or other interaction	Bogue (2015), Ogenyi et al. (2021), Dmytriyev et al. (2022), Eimontaite et al. (2022), Eimontaite et al. (2019), Hofer and Strohmeier (2019), Borboni et al. (2022), Eyam et al. (2021), Tsuji and Kohama (2022), Yeamkuan et al. (2022), Rato et al. (2022), Prati et al. (2022), Maurtua et al. (2017b), Marturi et al. (2019), Faibish et al. (2022), Ebrahimzadeh et al. (2019), Avalle et al. (2019), Abdelrahman et al. (2022)	18	263	14.61				
	Total		54	792	14.67				
Performance of actuating systems	Actuator, motion and trajectory control	Dufour et al. (2020), Zhao et al. (2022), Ye et al. (2020b), Wittmann and Rixen (2022), Vysocky et al. (2020a), Sidobre and Desornneaux (2019), Magyar et al. (2019), Li et al. (2019), Krämer and Bertram (2022), Kraemer et al. (2020), Chen et al. (2018), Liu et al. (2022a), Rojas et al. (2019), Rojas et al. (2020), Zhu et al. (2022), Palleschi et al. (2021), Ren et al. (2019a), Tadese et al. (2021), Zhen et al. (2022), Zhang et al. (2020), Zanchettin and Rocco (2017), Yan et al. (2024), Wu et al. (2021), Worsnopp et al. (2006), Tadese et al. (2022), Stradovnik and Hace (2022), Santos et al. (2022), Salvato et al. (2022), Roveda et al. (2020), Rouzbeh et al. (2018), Raviola et al. (2021b), Palmieri and Scoccia (2021), Moore et al. (2003), Mendoza-Trejo et al. (2019), Melchiorre et al. (2021), Mayer et al. (2020), Li et al. (2020d), Lan et al. (2020), Kana et al. (2021), Hu et al. (2019), Heredia et al. (2021), Guda et al. (2022), Gillespie et al. (2001), Ghidini et al. (2020), dos Santos et al. (2022), Darvish et al. (2018), Chuang et al. (2022), Cheng et al. (2021), Bi et al. (2008), Balatti et al. (2020), Ayvaci et al. (2022)	51	723	14.8	Colgate, J (4 publications)	Chinese Academy of Sciences (5 publications)	PRC (30 publications)	IEEE Robotics and Automation Letters (9 publications)
	Compliance control	Huang et al. (2019), Huang et al. (2020), Huang et al. (2021), Ubeda et al. (2021), Li et al. (2020c), Kim and Yang (2021), Dou et al. (2022), Zhang et al. (2021c), Zeng et al. (2019a), Xu and He (2022), Xiao et al. (2021), Pérez-Ubeda et al. (2020), Madsen et al. (2020), Fu and Zhao (2020), El Makrini et al. (2017), Abdallah et al. (2022)	16	318	19.87				
	Calibration	Pagani et al. (2021), Yun et al. (2022b), Kim et al. (2022), Pollák et al. (2020), Kolyubin et al. (2019), Semjon et al. (2020), Nadeau et al. (2019), Laliberté and Gosselin (2022)	8	71	8.87				
	Reducer and actuator	Fu and Cai (2022), Ayoubi et al. (2019), Ayoubi et al. (2020), Liu et al. (2021a), Yamada and Fujimoto (2021), Shin et al. (2022), Rouzbeh et al. (2019), Ostyn et al. (2022), Min and Song (2020), Min et al. (2019), Lee et al. (2018), Fernández et al. (2020)	12	115	9.58				
	Cobot architecture	Liu et al. (2021b), Cherubini et al. (2019), Romiti et al. (2022), Smrcek et al. (2022), Schou and Madsen (2017), Wojtynek et al. (2019), Surdilovic et al. (2003), Peshkin et al. (2001), Nguyen and Marvel (2022), Merckaert et al. (2018), Mendoza-Trejo and Cruz-Villar (2016), Krüger et al. (2006), Ibarguren et al. (2020), Hu et al. (2020a), Hu et al. (2020b)	15	403	26.87				
	Total		102	1,630	15.98				
Robot program generation	Programming by demonstration	Canfield et al. (2021), Ochoa and Cortesao (2022), Wang et al. (2019a), Wang et al. (2022a), Wang et al. (2023b), Svejda et al. (2022), Stenmark et al. (2018), Rozo et al. (2016), Müller et al. (2020), Iturrate et al. (2021), De Winter et al. (2019), Carfi et al. (2020), Al-Yacoub et al. (2021a), Ajaykumar et al. (2021), Wang et al. (2021), Soares et al. (2021), El et al. (2022), Halim et al. (2022), Safeea and Neto (2022), De Coninck et al. (2020), Zeng et al. (2019b), Mazhar et al. (2019), El et al. (2021), El Zaatari et al. (2021)	24	566	28.58	El Zaatari, S & Li, W (4 publications)	Coventry University (5 publications)	England (10 publications)	Robotics and Computer-Integrated Manufacturing (8 publications)
	Task-level/ skill-based	Schou et al. (2018), Giberti et al. (2022), Polverini et al. (2019), Herrero et al. (2017), Park et al. (2021b), Jezierski et al. (2019), Angleraud et al. (2021)	7	142	20.29				
	Other	El Zaatari et al. (2019), Zhang et al. (2022d), Rodamilans et al. (2016), Restrepo et al. (2020), Ren et al. (2019b), Prioli et al. (2022), Olivares-Alarcos et al. (2022), Lv and Qiao (2020), Kshirsagar et al. (2021), Ionescu and Schlund (2022), Ghadirzadeh et al. (2021), Fogli et al. (2022), Darvish et al. (2021), Dahl et al. (2022b), Cusano, 2023; Chen et al. (2021), Al-Yacoub et al. (2021b)	17	509	29.94				
	Total		48	1,217	25.35				

### 4.8 Advances for 2023 and 2024

Being aware that this paper presents an analysis of the scientific literature up to the end of 2022 in a fast-growing field, due to the constraints explained in Section 2.2, we present in this section some references, that give an idea of the trends in that domain for 2023 and 2024.

The International Federation of Robotics (IFR) (IFR, 2024) stated the top five global robotics trends in 2024: 1) AI and machine learning, 2) cobots in new applications, 3) mobile manipulators, 4) digital twins, and 5) humanoids. While trend 2 is about cobotics expanding in areas such as welding, trends 1, 3, and 4 can serve collaborative applications. Indeed, according to IFR, further cognitive collaboration with humans will be possible thanks to AI. Semeraro et al. (Semeraro et al., 2023) agree that machine learning has a great potential in HRI since it is a new way to develop cognitive models and behavioural blocks. IFR thinks that the possibility of mounting cobots onto mobile platforms generates new opportunities that will increase the demand for cobots. As shown in (Zafar et al., 2024), digital twins can serve collaborative applications by making them safer, through testing and optimizing the approach for HRI thanks to real-time data.

## 5 Conclusion and future research agenda

In cobot-related research, as the consulted scientific literature does not propose a comprehensive research agenda and lacks an extensive quantitative and qualitative analysis of the current state-of-the-art in cobotics, this paper has presented a scoping review and bibliometric analysis of the literature to investigate and reveal the development of cobotic research. Based on an analysis of 532 publications, retrieved from 2,195 records from the WoS database between 1996 and 2022, the study examined publication trends, leading journals, productive institutions, engaged countries, influential authors, and key topics. The study provides a macro-level guideline for cobotics researchers. The results demonstrate that, although the term “cobot” was first introduced in 1996 designating a passive manipulator, documented research activity in the field of industrial robots designed for collaboration with humans has become more regular from 2016 and has experienced exponential growth since then. The “Politecnico di Milano” is identified as the leading institution in terms of the number of publications, and the journal “Robotics and Computer-Integrated Manufacturing” is the primary source of publications. Professors Vidoni and Vanderborght are recognized as the most productive authors based on publication count. The contribution of countries was evaluated using two additional indices: the number of articles *per capita* and the number of articles *per capita* GDP. While Italy leads in total publications, Denmark has the highest proportion of its population engaged in cobotics research, and the PRC ranks first in terms of financial support for cobotics research. Furthermore, this paper provides a univocal categorization that includes deployment of cobots, safety in cobotics for industry, human-robot tasks allocation, human-robot interaction, performance of actuating systems, and robot program generation. This classification helps cobotic researchers in different fields understand research developments and trends, identify opportunities for collaboration, and select appropriate journals for publication. The most active research topic categories are, in order of importance: “safety in cobotics for industry”, “deployment of cobots”, and “performance of actuating systems.” In the latter, the “actuator, motion and trajectory control” sub-category was found to be the most popular among cobotic researchers.

Although this paper reviewed as many relevant papers as possible, the results are limited to documents indexed in the WoS database. Consequently, there may be other publications not indexed in this database, which could introduce inaccuracies in the results. Nonetheless, despite cobotics being a highly multidisciplinary research field, the co-authorship analysis reveals that there is still a limited international collaborative community actively engaged in cobotic research. Further research in the cobotics field could benefit from more international collaboration with multidisciplinary experts. Also, although the results showed that deploying cobots is one of the most prominent research topics in the field of cobotics, and cobot applications are rapidly increasing in industrial sectors such as assembly, packaging, and surface treatment, there remains significant potential for exploring other opportunities. Designing, and deploying cobots in new use cases can greatly advance the field of cobotics research. As clearly presented in this paper, there is an increase in research activity but not a diversification of research topics. It may be time to explore new research topics aligned with current industry challenges and societal needs: for example, social responsibility is not effectively addressed, which could be an important future research direction. Literature on cobots can also be reviewed and analyzed through emerging trends such as sustainability and Artificial Intelligence (AI). Therefore, conducting systematic reviews and bibliometric analyses on sustainability in cobots or the application of AI to cobots could be a promising avenue for future research.

## Data Availability

The original contributions presented in the study are included in the article/supplementary material, further inquiries can be directed to the corresponding author.
